# Reconstruction of genome-scale metabolic models for 126 human tissues using mCADRE

**DOI:** 10.1186/1752-0509-6-153

**Published:** 2012-12-13

**Authors:** Yuliang Wang, James A Eddy, Nathan D Price

**Affiliations:** 1 Institute for Systems Biology, 401 Terry Ave N, Seattle, WA, 98109, USA; 2 Department of Chemical and Biomolecular Engineering, University of Illinois, Urbana, IL, 61801, USA; 3 Department of Bioengineering, University of Illinois, Urbana, IL, 61801, USA

**Keywords:** Automated metabolic network reconstruction, Brain, Cancer metabolism, Tissue-specific metabolic model, Constraint-based modeling

## Abstract

**Background:**

Human tissues perform diverse metabolic functions. Mapping out these tissue-specific functions in genome-scale models will advance our understanding of the metabolic basis of various physiological and pathological processes. The global knowledgebase of metabolic functions categorized for the human genome (*Human Recon *1) coupled with abundant high-throughput data now makes possible the reconstruction of tissue-specific metabolic models. However, the number of available tissue-specific models remains incomplete compared with the large diversity of human tissues.

**Results:**

We developed a method called metabolic Context-specificity Assessed by Deterministic Reaction Evaluation (mCADRE). mCADRE is able to infer a tissue-specific network based on gene expression data and metabolic network topology, along with evaluation of functional capabilities during model building. mCADRE produces models with similar or better functionality and achieves dramatic computational speed up over existing methods. Using our method, we reconstructed draft genome-scale metabolic models for 126 human tissue and cell types. Among these, there are models for 26 tumor tissues along with their normal counterparts, and 30 different brain tissues. We performed pathway-level analyses of this large collection of tissue-specific models and identified the eicosanoid metabolic pathway, especially reactions catalyzing the production of leukotrienes from arachidnoic acid, as potential drug targets that selectively affect tumor tissues.

**Conclusions:**

This large collection of 126 genome-scale draft metabolic models provides a useful resource for studying the metabolic basis for a variety of human diseases across many tissues. The functionality of the resulting models and the fast computational speed of the mCADRE algorithm make it a useful tool to build and update tissue-specific metabolic models.

## Background

Metabolic dysfunction has been implicated in a wide variety of human diseases such as obesity, diabetes, inborn errors of metabolism, neurodegenerative diseases, and cancer. The recent reconstruction of genome-scale models of human metabolism [1,2] provides an important biochemical basis for systems analysis of metabolic related aspects of human physiology and pathology [3]. Such systems approaches are critical, as metabolism itself is a molecular transformation process where numerous metabolic pathways are inextricably interlinked [4]. However, the human body consists of many distinct tissues and cell types, each only expressing a fraction of the metabolic genes encoded within the genome [5]. Additional variability arises from environmental conditions and external stimuli. None of this variation can be fully accounted for with only the generic human metabolic model. Considering the context—e.g., genomic, anatomical, environmental, or temporal—under which a subset of the genome-scale biochemical network operates is therefore essential to understanding the molecular basis for many human diseases.

The importance of tissue-specific context in disease is evident from distinct metabolic profiles of cancers arising from different tissues. For example, it has been experimentally demonstrated that *MYC* oncogene-induced liver tumors show increased glutamine *uptake*, while *MYC*-induced lung tumors show glutamine *secretion*[6]. Another study showed that while lactate dehydrogenase A is important for breast carcinoma, neuroblastoma, and B-cell tumor cells, it is dispensable for *MYC*-induced lymphomagenesis [7]. Similar results were observed for phosphoglycerate dehyrogenease in breast cancer and melanoma [8,9] versus *MYC*-induced lymphomagenesis [7]. Importantly, cancer metabolism in general also operates in unique environmental and signaling contexts compared to normal physiology and metabolic diseases such as obesity and diabetes [4].

The context in which a metabolic network operates can be viewed at multiple scales, all of which can be dependent on one another. The broadest level typically associated with metabolic models is genomic context—i.e., the full enzymatic capability encoded in the genome. Since the genome is the starting point from which to construct any generic organismal model, we will not consider it further here. A more critical contextual consideration for genome-scale models in higher organisms—especially in human tissues—is the subset of metabolic enzymes that are being expressed (e.g., represented in the transcriptome) at a given time. The transcriptional regulatory state governs which subset of metabolic enzymes and pathways are active, and manifests as either (i) the specific expression program for a tissue or cell type; or (ii) the tissue or cellular response to intracellular or environmental conditions. The ideal strategy for modeling such contextual differences would be the integration of a generic, genome-scale model (e.g., *Human Recon 1*[1]) with a detailed, context-specific transcriptional regulatory network (TRN), including signaling events that relay cues from the cellular microenvironment. However, as these TRNs cannot yet be comprehensively and accurately reconstructed and modeled in human cells, recent efforts have turned to employing context-specific expression data to create models that are representative of active metabolism in specific human tissues and cell types either across a wide range of experimental conditions or under a particular condition [10-19].

For clarity, we will henceforth delineate “*tissue*-specific” as meaning the representative active metabolic network for a tissue (e.g., liver, brain), and “*condition*-specific” as specific network states (e.g., hypoxia, drug treatment) of *tissue*-specific models. We also note that when higher resolution data is available, tissue-specific models can be further discretized into region or cell type specific models (e.g., different regions of brain, different neuron subtypes). Tissue-specific models are generally more desirable than condition-specific for predictive modeling, because they retain the flexibility and redundancy inherent in the metabolic network; specific conditions can subsequently be simulated directly by defining model constraints. Generating condition-specific models can still be highly useful, especially when coupled with experimental data for testing and validation; methods such as iMAT [10] and GIMME [17] have been used successfully to estimate the metabolic state of tissues under particular pathophysiological conditions. While the need for tissue-specific metabolic models is strong, the available number remains small. Importantly, significant knowledge and data is required to reduce a generic model to a tissue-specific model with enough rigors to allow for different condition-specific capabilities. Computational tools that can more rapidly generate tissue models that can represent a spectrum of physiological conditions will be highly useful for investigating metabolic dysfunctions in various diseases.

The Model Building Algorithm (MBA), a current state-of-the-art computational method to build tissue-specific metabolic models, has been used to build liver, generic cancer, and two cancer cell line metabolic models thus far[14-16]. The resulting models have been used to predict potential drug targets and improve metabolic flux predictions [14-16]. While a core set of high-confidence reactions in MBA is determined based on gene expression or literature evidence, the ranking and inclusion of non-core reactions is based on iterative model simulation for many different random reaction orderings. Notably, the random sampling in MBA—on the order of 1000 iterations in published studies—is limited in its coverage of the large space of possible orderings, potentially affecting the accuracy of the tissue-specific model. While this problem is mostly avoided by a stringent requirement in MBA for model consistency (i.e., all reactions in the final tissue-specific model must be capable of carrying flux), a more deterministic and simulation-independent ranking of non-core reactions would serve to dramatically speed up model construction time.

We have developed a method called metabolic Context-specificity Assessed by Deterministic Reaction Evaluation (mCADRE) that leverages gene expression evidence, network structure, and metabolic function to construct context-specific models in an automated, deterministic, and high-throughput fashion. Similar to MBA, mCADRE emphasizes the inclusion of a high-confidence core set of reactions from a generic genome-scale model, based on tissue-specific expression evidence. Non-core reactions are explicitly ranked according to their own expression evidence as well as weighted connectivity to other reactions in the network, and then sequentially removed in the inverse order of this ranking. The decision whether to confirm or reject each removal is determined by the consequent flux capacity of core reactions, as well as a universal test of metabolic functionality. To evaluate the performance of our method, we reconstructed a new liver model and compared results to liver models built by MBA: mCADRE was able to achieve similar coverage of high evidence reactions, improved metabolic functionality, and dramatic speed up. The deterministic decision making in mCADRE, coupled with an automated pipeline of data collection and processing, enables researchers to efficiently generate accurate and robust initial models from publicly available expression data.

As a demonstration of mCADRE’s capabilities, we leveraged data from the Human Gene Expression Barcode Project [20] to automatically reconstruct draft genome-scale metabolic models for 126 human tissues and cell lines, collectively called the Tissue-Specific Encyclopedia of Metabolism (TSEM). All 126 metabolic models (Additional file 1), mCADRE codes and input data (Additional file 2) are available at [21] and are also available in the supplemental materials. We identified many amino acid metabolic pathways as enriched in 30 brain tissue models in TSEM, which agrees with the known role of amino acids in neurotransmitter metabolism. By comparing tumor and normal metabolic networks in TSEM, we also identified pathways with known roles in tumor metabolism. In particular, we identified part of the eicosanoid metabolic pathway as a potential selective target against tumor tissues. Further analysis of metabolic networks in TSEM, especially through integration with regulatory networks and various omics data, may offer novel insights of the metabolic aspects of various diseases.

## Results and discussion

### Method overview and advantageous features of mCADRE

**Figure 1:**
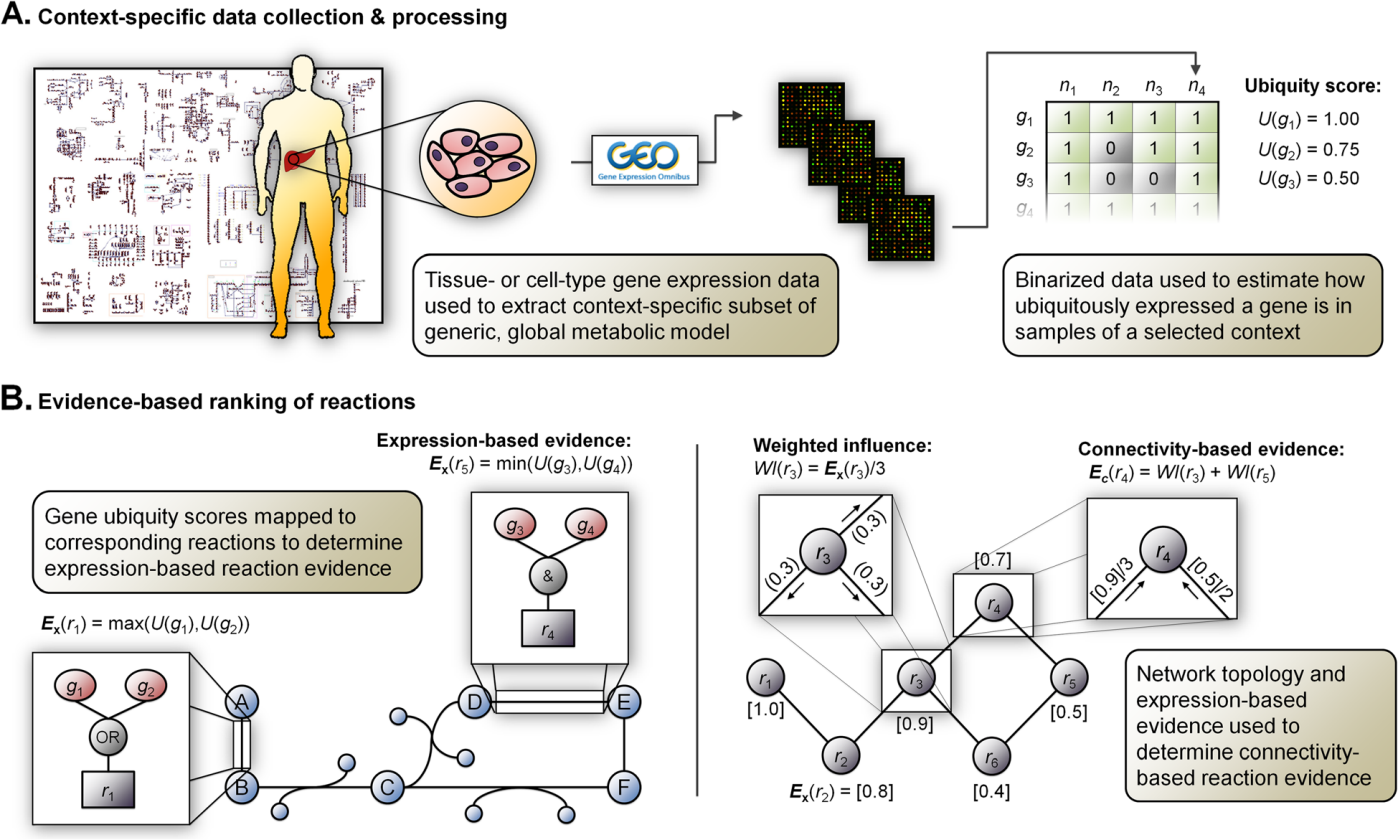
**mCADRE method overview.** (**A**) After binarizing context-specific input data, we quantify how often a gene is expressed across samples of the same tissue; this is the ubiquity score *U*(*g*) for each gene *g*. (**B**) From ubiquity scores, we calculate the *expression-based evidenceE*_*x*_ for each gene-associated reaction. Reactions with sufficiently high *E*_*x*_ are defined as the core reaction set and are included in the tissue-specific model. To deterministically rank non-core reactions with low to moderate expression-based evidence, we introduce a network topology metric to calculate *connectivity-based evidenceE*_c_.

mCADRE builds a tissue-specific model from a generic human metabolic model [1] based primarily on gene expression data and metabolic network topology (Figure 1). Like MBA, we define a core set of reactions that should be present and active (i.e., able to carry flux) in the tissue model (we have implemented an adapted version of the *checkModelConsistency* module described in Jerby et al. to identify blocked reactions). The set of core reactions are determined from gene expression, and non-core reactions are evaluated and ranked according to a combination of expression and connectivity evidence (detail description in Methods). To help ensure the basic functionality of the tissue-specific models, mCADRE includes a metabolic function test in the model building process. Specifically, the *checkModelFunction* module tests the ability of the current model to produce key metabolites from glucose, based on criteria previously used to universally evaluate such models [18] (see Methods and Additional file 3: Table S1 for further detail). This list can be customized based on literature evidence or metabolomics data (when available) to include tissue-specific metabolites or known capabilities of the tissue or cell type. We sequentially prune non-core reactions from the generic model in the determined order, provided that removal does not affect fluxes through the core reaction set or production of key metabolites from glucose. The former requirement is waived when removing non-core reactions whose associated genes are not expressed in *any* tissue samples. For each reaction removed from the generic model, all resulting inactivated reactions are also removed.

#### Allowing for a flexible core reaction set increases tissue-specificity of metabolic pathways

In addition to the core set of reactions (whose associated genes are expressed in many tissue samples), mCADRE defines a negative set of reactions whose genes are not expressed in any tissue samples. In this case, when expression evidence strongly suggests that a reaction should not be included, we relax the constraint described above to also allow for removal of any consequently inactivated core reactions. The non-expressed reaction is removed, along with all reactions that can no longer carry flux, only if the ratio of resulting inactivated core reactions to inactivated non-core reactions is smaller than a specified ratio. This parameter governs the sensitivity versus specificity of the final tissue model: a lower ratio cutoff leads to inclusion of more reactions with strong positive evidence, while a higher cutoff leads to removal of more reactions with strong negative evidence. It is important to note the difference between non-expressed reactions (expression evidence strongly suggest the absence of such reactions) and non-gene associated reactions (expression evidence not applicable). Non-gene associated reactions include spontaneous reactions and reactions catalyzed by enzymes not annotated to genes yet. Non-gene associated reactions are not included in the negative set, and no core reactions are allowed to be removed when mCADRE tries to remove these reactions.

**Figure 2:**
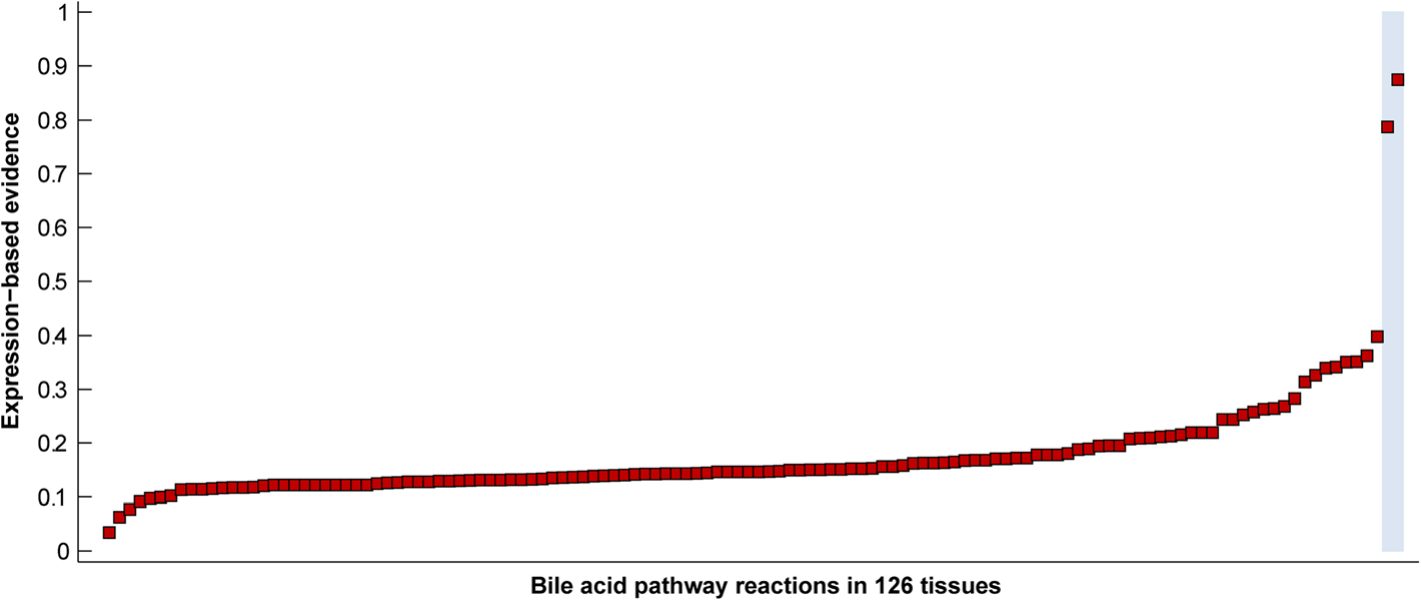
**Expression-based evidence for the bile acid synthesis pathway across 126 tissues.** Red squares indicate the average expression-based evidence of all bile acid reactions in the tissue. Normal liver and liver cancer tissues, known to synthesize bile acids, are highlighted in blue.

The utility of allowing for a flexible core can be seen with the bile acid biosynthesis pathway in the liver. Although many cell types express several enzymes in the bile acid pathway, the complete pathway is present only in the liver [22]. The tissue-specificity of this pathway is also supported by microarray data: among 126 tissues, we found that almost all bile acid synthesis reactions have strong evidence of activity in the liver, but not in other tissues (Figure 2). However, a few reactions in this pathway have strong evidence in non-liver tissues (e.g., cerebral cortex). If a "hard" core were to be enforced—i.e., requiring all reactions with expression evidence above a threshold to carry flux and remain in the tissue model—most reactions in the bile acid synthesis pathway would be included in these tissues, even though most reactions in the pathway lack expression evidence. When we allowed for a flexible core, only liver and liver cancer models included almost complete bile acid synthesis pathways (85% of pathway reactions present), while most other tissues did not have reactions from this pathway. In contrast, when using a hard core, most models included a majority of bile acid reactions (60%~80% of pathway reactions), conflicting with known tissue specificity. For cerebral cortex, for example, 70% or 5% of the bile acid synthesis pathway is computed as present when using a hard or flexible core, respectively, supporting the importance of including the flexible core in the mCADRE approach. This result is achieved when the inactivated core to non-core reaction ratio is set at 0.33, and sensitivity analysis shows that the resulting models are robust to the precise selection of the cutoff ratio (Additional file 3: Table S2).

There are 541 and 844 confidently positive (expressed in more than 50% of tissue samples) and negative (not expressed in any tissue sample) metabolic reactions in the cerebral cortex, respectively. In reconstructing the final model, tradeoffs must be made between these sets. For example, 49 confidently negative reactions are essential for basic functionality of the model (glycolysis, TCA cycle, pentose phosphate pathway). To remove all the other 795 (94%) confidently negative reactions, 185 (34%) confidently positive reactions would be removed. On the other hand, to include all 541 confidently positive reactions, 231 (27%) confidently negative reactions have to be included. At ratio 0.33, as shown in the Additional file 3: Table S2, 28 (5%) confidently positive reactions were removed, while 672 (80%) confidently negative reactions were removed.

There are 172 remaining reactions whose associated metabolic genes are not expressed in any cerebral cortex microarray samples but still retained in the model to complete necessary functionalities. For 130 of the 172 reactions, protein-level staining evidence is available in the Human Protein Atlas (HPA) [23]. Among these 130 reactions, according to gene-reaction mapping, 21, 69, 36, and 4 reactions have negative, weak, moderate, and strong protein staining evidence, respectively. Therefore, more than 80% of confidently negative reactions based on transcriptomic data that were retained for functionality considerations by mCADRE do in fact have some proteome-level evidence. This can either be caused by limitations of the microarray gene expression measurements, or potential effects of post-transcriptional regulation of the corresponding metabolic genes.

Compared with enforcing a strict inclusion of all the core reactions with the ability to carry metabolic flux, 28 core reactions are removed if when we allow a flexible core reaction set in building the cerebral cortex model. These reactions are removed because many non-expressed reactions need to be included in the model for these 28 reactions to carry flux. Among the 28 reactions, there are 5 UDP-glucuronosyltransferase reactions encoded by UTG1A8 and UTG1A10. UDP-glucuronosyltransferase reactions occur mainly in the liver and intestines, and neither UTG1A8 nor UTG1A10 are expressed in the brain in general or the cerebral cortex in particular [24,25]. Three reactions in bile acid synthesis, all associated with SCP2 (sterol carrier protein 2) are also removed. Sterol carrier protein 2 shows negative staining in cerebral cortex neuronal and glial cells according to HPA. However, according to the input microarray data from the Gene Expression Barcode, all UDP-glucuronosyltransferase reactions and reactions associated with sterol carrier protein 2 have high expression-based evidence (0.65 and 1, respectively). In these two examples, mCADRE correctly removed reactions for which many other reactions with little expression evidence are needed to carry flux. In summary, mCADRE is designed with the goal of balancing strongly positive and negative expression evidence. Inconsistent reactions based on gene expression data (negative reactions retained in the model and core reactions removed from the model) for each of the 126 tissue-specific models are included in Additional file 4 to facilitate further manual curation.

#### mCADRE significantly reduces computation time to generate context-specific models

The novel reaction ranking scheme based on three criteria (gene expression, network connectivity, and literature-supportted reaction confidence level encoded in *Human Recon 1*) enables mCADRE to perform a single optimized iteration to infer a tissue-specific model from the generic human metabolic map. In contrast, MBA determines whether to retain non-core reactions through a large number of random iterations (typically ~1000) to account for the effects of the order in which reactions are removed [14]. The order of reaction removal remains influential in mCADRE—e.g., redundant reactions can be removed interchangeably with equal effects on core reactions, but whichever reaction is pruned first mandates the retention of the latter. However, mCADRE leverages gene expression, topology, and literature evidence to directly determine ordering in a quick deterministic fashion, avoiding random iterations. Each iteration in MBA involves Flux Variability Analysis (FVA [26]; maximization and minimization of all reactions to calculate flux capacity), which amounts to on the order of 10,000 separate optimizations. This computational complexity limits not only the throughput of reconstructing metabolic models, but potentially their reproducibility: as the full solution space of ordered reaction removals is extremely large, the 1000 permutations sampled by MBA necessarily represent only a tiny fraction of all possibilities. The stringent requirement in MBA that final tissue-specific models be consistent (i.e., contain no gaps) enforces the inclusion of many lower-evidence reactions, and leads to mostly similar models from run to run. Still, even with a heuristic speed-up [14] or the efficient fastFVA algorithm [27], one iteration of MBA takes ~10 hours on a single 2.34 GHz CPU with 4G RAM using the open source glpk solver. The whole MBA reconstruction process, with ~1000 iterations, would therefore take on the order of ~10,000 CPU-hours. mCADRE dramatically improves the computational speed via deterministic evidence-based evaluation of reactions, requiring only ~10 CPU-hours under the same configuration. With the IBM CPLEX solver (free for academic institutions), the model reconstruction time was further reduced to 4 hours.

Manual curation of metabolic models often continues over several iterations of simulation-based hypothesis generation, experimental validation, and model refinement to improve quality and predictive accuracy. Such iterative curation is also important in computational model reconstruction, especially when new and better (i.e., more comprehensive, more sensitive, higher resolution) data becomes available. New technologies such as RNA-seq provide unprecedented characterization of the transcriptome [28] with a much lower detection limit than microarray. As RNA-seq data become available for a variety of tissues and cell types [29], it is important that corresponding models are updated to better reflect the metabolic capacity corresponding tissues: metabolic genes expressed at low levels may be regarded as not expressed by microarray and excluded from metabolic models. Because mCADRE reduces the computational time of model reconstruction almost 1000 fold, it is much more convenient to build or update a large collection of tissue-specific models when new data are released.

### Coverage-based and functional validation of a mCADRE-constructed liver model

As initial validation of the mCADRE method, we used the algorithm to reconstruct a liver model (*liver*CADRE, Additional file 5) and compared it to the liver model in the original MBA publication [14] (henceforth referred to as *liver*MBA), as it is the best characterized MBA-generated tissue model to date. We built the *liver*CADRE model based on 23 normal liver microarray samples (Additional file 3: Table S3). Notably, both mCADRE and MBA result in consistent final tissue models, so all liver model reactions examined are able to carry flux (Table 1). While *liver*CADRE includes 1763 reactions to the 1826 in *liver*MBA, the two models share 1473 reactions, a significant overlap (under hypergeometric distribution, the probability of observing 1473 or more overlapping reactions is 1.54×10^-12^; N = 2469, the total number of flux-carrying reactions in *Recon 1*); these overlapping reactions constitute over 80% of all reactions in each model, and thus substantial convergence between the approaches, establishing confidence for the quality of models generated with mCADRE. To more directly evaluate the performance of mCADRE and MBA for generating new tissue-specific models, we also used MBA to build a model from our liver expression training data (Additional file 3: Table S4); *liver*CADRE exhibited similar or better coverage and increased functionality in comparisons with the new MBA model built with the same training data.

**Table 1:** Summary of the mCADRE liver model and the original MBA model

	***liver*CADRE**	***liver*MBA**
Total reactions	1763	1826
Gene-associated reactions	1194	1167
Total genes	1267	1333
Total metabolites	1402	1360

#### mCADRE-constructed liver model improves coverage of highly expressed genes and proteins

**Figure 3:**
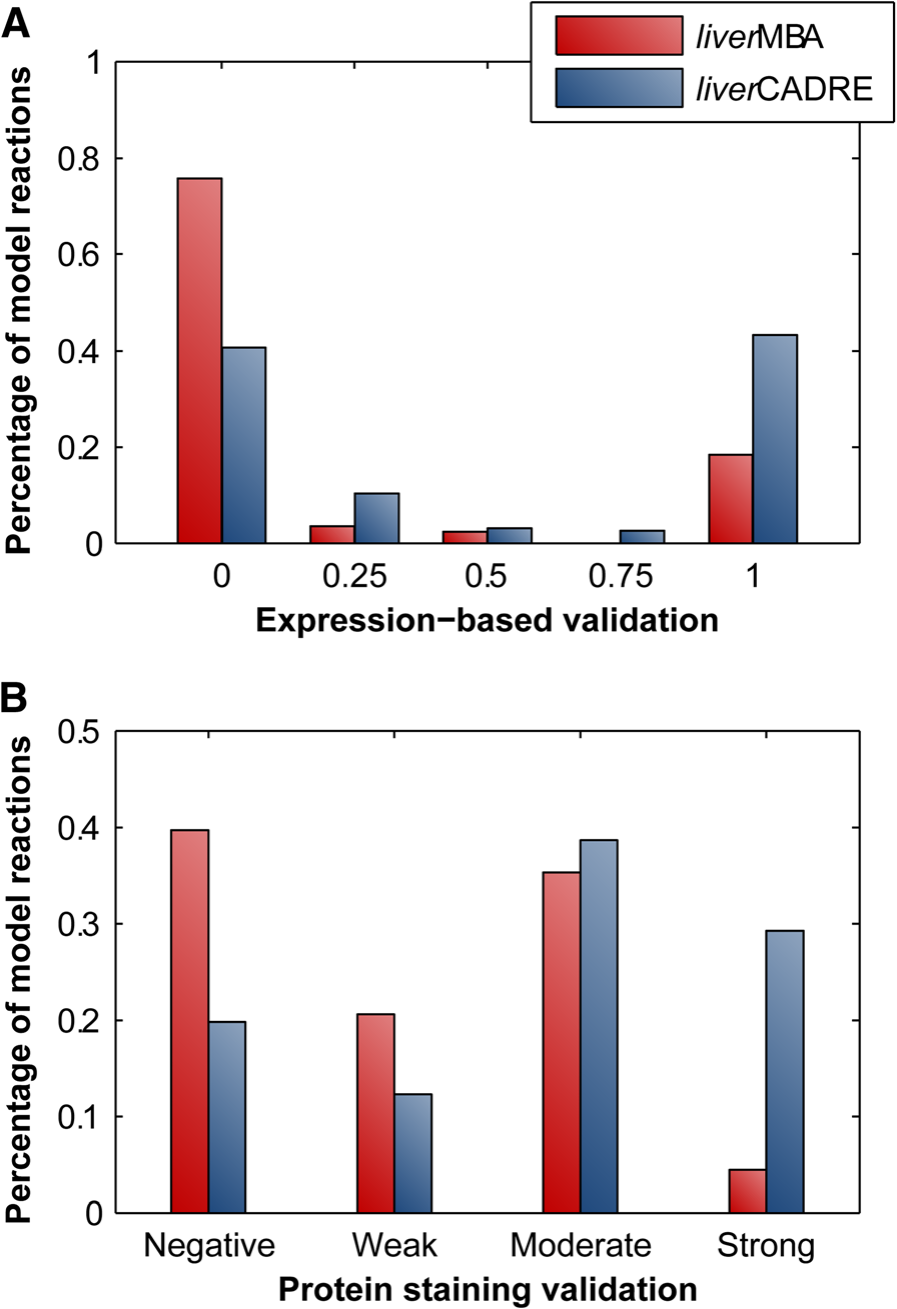
**Coverage-based comparison of mCADRE and MBA liver models.** (**A**) Expression-based validation was calculated from an independent microarray data set for reactions unique to *liver*MBA or *liver*CADRE. (**B**) Protein staining data from Human Protein Atlas was mapped to reactions in each model.

While the two models share most reactions, we chose to further explore the gene-associated reactions unique to each model. There are 194 and 169 gene-associated reactions unique to *liver*CADRE and *liver*MBA, respectively. For each set of reactions, we first examined the coverage of highly expressed metabolic genes in an independent data set (test data set, Additional file 3: Table S5), not used in building either liver model and based on a different microarray platform than any of the training data used by mCADRE. We assume that reactions with strong expression-based *validation* (whose associated metabolic genes are most ubiquitously expressed across new tissue-specific samples) are more likely to be present in the liver. The set of gene-associated reactions unique to the mCADRE model have higher expression-based validation score than gene-associated reactions unique to *liver*MBA (Wilcoxon rank sum test p-value: 6.02 × 10^-9^; Figure 3A). *liver*CADRE includes more reactions with strong expression-based validation than the MBA model (46% vs. 18%) and fewer reactions with poor to no gene expression-based validation than *liver*MBA (47% vs. 76%; Figure 3A).

As mRNA and protein levels are only moderately correlated in mammalian cells [30], we also compared coverage of the two liver models at the protein expression level. We collected protein staining data from the Human Protein Atlas (HPA) [23] for 560 metabolic genes in *Recon 1* (Additional file 3: Table S6). Protein staining strength is divided into four levels by HPA: strong, moderate, weak, and negative. We mapped the HPA data to reactions according to gene-reaction associations, and assigned each reaction a score, based on the staining strength of its associated metabolic gene. As shown in Figure 3B, 29% of gene-associated reactions unique to *liver*CADRE have strong protein staining support, and 20% have negative staining support. In comparison, only 4% of reactions unique to *liver*MBA have strong protein staining support, while 40% have negative protein staining support. Moreover, gene-associated reactions unique to *liver*CADRE collectively have significantly higher scores than *liver*MBA (Wilcoxon rank sum test *p*-value: 1.25 × 10^-5^).

#### mCADRE-based liver model is functionally comparable to the existing liver model

The liver plays a major role in metabolism and carries out important metabolic functions such as gluconeogenesis, triglyceride synthesis, amino acid degradation, and ammonia and ethanol detoxification. We investigated the ability of the two liver models to carry out these hepatic metabolic functions; the details of each metabolic function simulation are described in Methods. We found that the two models are functionally comparable (Table 2), which is expected given that they share a large percentage of reactions. Both models can detoxify ammonia and ethanol; both can simulate gluconeogenesis from physiologically important substrates such as pyruvate, lactate, alanine and glutamine; and both models can degrade most amino acids and produce urea as byproduct. While *liver*MBA can degrade more amino acids and generate glucose from a broader range of glucogenic substrates, only *liver*CADRE is able to synthesize triglyceride from glucose and fatty acids. Triglyceride synthesis is a major hepatic function underlying blood glucose and lipid homeostasis — the ability to simulate this function *in silico* enables the investigation of liver metabolic network states in normal and pathological conditions such as obesity and fatty liver disease. While *liver*MBA includes over 700 reactions manually curated to be active in the liver [14], no such curation was done to build *liver*CADRE. *liver*CADRE also outperforms the new MBA liver model built with the same training data in liver metabolic function tests (Additional file 3: Table S4). Detailed results of the liver function simulations can be found in Additional file 3: Table S7-S9.

**Table 2:** Results of hepatic metabolic function simulations

**Functional tests**	***liver*CADRE**	***liver*MBA**
gluconeogenesis	13/21	19/21
Triglycerol synthesis	1/1	0/1
Amino acid degradation	19/20	20/20
Ammonia detoxification	1/1	1/1
Ethanol detoxification	1/1	1/1
Nucleotide synthesis	8/8	4/8

The liver is also able to regenerate after injury, which involves the synthesis of biomass precursors such as nucleotides, amino acids, and lipids. A biomass reaction was added to both models (after construction) that accounts for the growth requirement of amino acids, nucleotides, lipids, and other metabolites (Additional file 3: Table S10 and Methods), and we tested the ability of the two models to grow *in silico* in RPMI 1640 tissue culture medium conditions (Additional file 3: Table S11). The *liver*CADRE model was able to simulate growth without further manual curation, while *liver*MBA lacked this capability. Further analysis identified that *liver*MBA could not grow because it contained no reactions in the inosine monophosphate (IMP) pathway, and therefore could not produce purines. As *de novo* purine synthesis primarily occurs in the liver [22], this lack of this capability represents a metabolic gap in *liver*MBA. Moreover, many membrane phospholipids such as phosphatidic acid, phosphatidylethanolamine, phosphatidylcholine and phosphatidylserine are derived from triglyceride. As *liver*MBA cannot produce this metabolite, the production of these glycerophospholipids is consequently blocked. This demonstrated the importance of the metabolic function test in mCADRE, as it ensures the basic functionality of the resulting model and may save substantial *post hoc* manual curation.

### mCADRE for high-throughput model generation

After verifying that our new liver model could show similar or better coverage and functionality when compared to state-of-the-art models and algorithms, we next took advantage of the automated and computationally efficient nature of mCADRE to generate a large collection of tissue and cell type specific metabolic models. The Gene Expression Barcode project previously collected, annotated and binarized microarray data for 126 human tissues and cell lines on the Affymetrix U133Plus2 platform [20]. We used these binarized microarray data sets as input evidence in mCADRE to extract individual models from the generic *Recon 1*, thereby establishing a Tissue-Specific Encyclopedia of Metabolism (TSEM). This effort provides the most comprehensive mapping to date of human tissue-specific metabolic networks, and for many of the 126 tissues or cell types, this represents the first time a genome-scale metabolic model has been built.

#### Tissue-specific Encyclopedia of Metabolism enables global analysis of human tissues

The Tissue-Specific Encyclopedia of Metabolism (TSEM) includes 26 tumor tissues and cell lines, and 18 of these tumor tissues also have corresponding normal tissue models. It also contains metabolic models of 30 different brain tissues, many of which are affected in various neurological diseases. A full list of the 126 tissues and the corresponding microarray data can be found at [20] and [31]. All new metabolic models already include several important features for *in silico* simulation of cellular behavior: they have functional central metabolic pathways (glycolysis, TCA cycle, pentose phosphate pathway), can synthesize non-essential amino acids from glucose, synthesize nucleotides via *de novo* or salvage pathways, have functional fatty acid synthesis pathway (from acetyl-CoA to palmital-CoA), and can synthesize key membrane lipids. These functionalities are a result of the basic universal metabolic function test integrated into mCADRE, which can be further customized to reflect the specific capabilities of individual tissues and cell types. The latest version of these models, as well as additional models built with latest data (e.g., RNA-seq) can be downloaded from [21]. To facilitate further manual curation, inconsistent reactions (core reactions that removed during model reconstruction and non-expressed reactions retained in the final tissue model) for each model are included in the Supplementary Material as well as in the above website.

**Figure 4:**
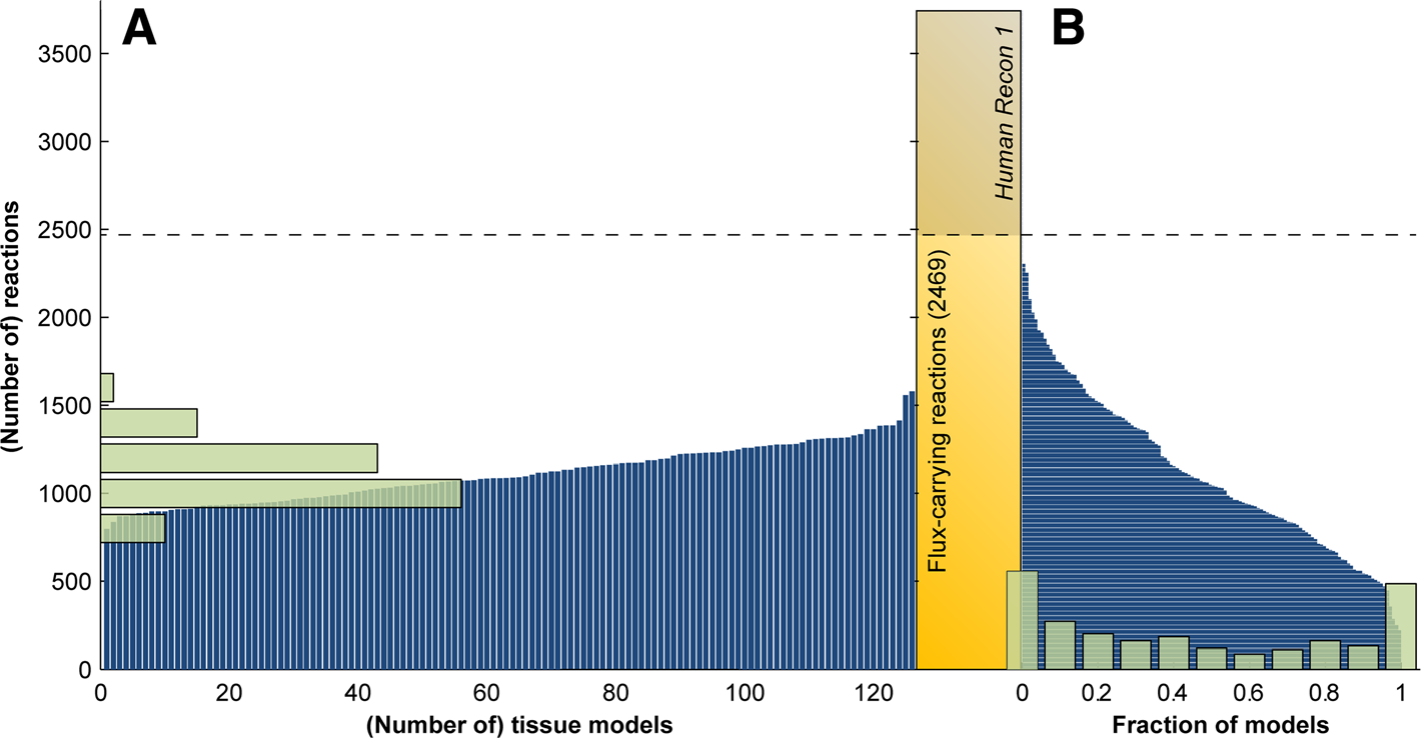
**Number and distribution of reactions in TSEM models.** (**A**) Vertical blue lines indicate the number of reactions in each tissue model; green bars show the size distribution across TSEM models. (**B**) Horizontal blue lines indicate the fraction of models in which each *Recon 1* reaction is included; green bars show the frequency distribution across reactions.

With this comprehensive set of tissue-specific draft metabolic models, we can start to evaluate global properties of these networks and their relationship to human metabolism in the body (Figure 4). Models in the TSEM contain 1161 reactions on average (47% of flux-carrying reactions in *Recon 1*), with most models ranging from 1000 to 1300 reactions (Figure 4A). The smallest model is neutrophiles, which included 826 reactions. The largest models are liver tumor, normal liver and kidney, which included 1550, 1530 and 1416 reactions respectively. This is expected as the liver and kidney are among the most metabolically active tissues in the human body. There are 2311 reactions that appeared in at least one of the 126 context-specific models, representing 93% of the flux-carrying reactions in *Recon 1* (Figure 4B); 600 reactions appear in at least 90% of the 126 models, and 546 reactions appear in at most 10% of the models.

#### Distributions of TSEM model reactions correspond to known features of brain and tumor tissues

We identified pathways that are enriched in brain tissue models—i.e., pathways with more reactions present in normal brain tissues than in normal non-brain tissues (Table 3). Among the pathways most enriched for completeness in brain (compared to normal non-brain tissues) were taurine and hypotaurine metabolism, aromatic amino acid biosynthesis, cysteine metabolism, alanine and aspartate metabolism, glutamate metabolism, and valine, leucine, and isoleucine metabolism. This makes sense, as many amino acids are either neurotransmitters or intermediates in neurotransmitter synthesis. The brain is also known to contain higher concentrations of long-chain polyunsaturated fatty acids (PUFAs) than most other tissues, and both cerebral endothelium and astrocytes elongate and desaturate precursors of the long-chain PUFAs [32,33]. As such, it is not surprising to see that the fatty acid elongation pathway includes significantly more reactions in brain tissues than non-brain models. Pathways better enriched in brain tissue models are in agreement with known brain-specific metabolic functions, demonstrating the quality of these models.

**Table 3:** ***Recon 1 *metabolic pathways differentially represented in brain and non-brain normal tissues**

**Pathways**^**a**^	**% complete in brain models**	**% complete non-brain models**	**Rank sum p-value**
Taurine and hypotaurine metabolism	66%	35%	4.14E-08
Fatty acid elongation	67%	37%	1.69E-09
Tyr, Phe, Trp Biosynthesis	77%	56%	4.96E-02
Salvage Pathway	94%	81%	2.26E-02
Cysteine Metabolism	50%	37%	3.61E-03
Alanine and Aspartate Metabolism	73%	61%	2.41E-09
Glutamate metabolism	86%	76%	1.59E-06
Butanoate Metabolism	32%	23%	9.34E-03
Valine, Leucine, and Isoleucine Metabolism	69%	61%	4.00E-02
Transport, Nuclear	33%	25%	2.05E-11

^a^Pathways are sorted by difference in percentage of reactions present in brain vs. non-brain tissues.

We also identified pathways enriched in the 18 tumor tissues compared to their 17 corresponding normal tissues (including two different tumors that arise from the same normal tissue; Table 4), including folate metabolism, eicosanoid metabolism, fatty acid activation and nucleotide metabolism. Folate metabolism is necessary for *de novo* nucleotide synthesis. The enrichment of reactions for this pathway—as well as the nucleotides pathway—in tumor tissue models makes sense because nucleotide synthesis is more active in proliferating tumor cells, and many enzymes in nucleotide synthesis are classical chemotherapy targets.

**Table 4:** ***Recon 1 *metabolic pathways differentially represented in tumor and normal tissues**

**Pathways**^**a**^	**% complete in tumor models**	**% complete in normal models**	**Rank sum *p*-value**
Folate Metabolism	50%	27%	2.8E-03
Eicosanoid Metabolism	34%	13%	6.6E-04
Fatty acid activation	91%	81%	1.8E-02
Tryptophan metabolism	17%	10%	1.2E-02
Transport, Lysosomal	17%	11%	7.8E-03
Nucleotides	69%	63%	1.9E-04
Aminosugar Metabolism	56%	53%	4.8E-02
Transport, Mitochondrial	25%	23%	3.4E-02
Sphingolipid Metabolism	13%	12%	3.2E-02

^a^Pathways are sorted by difference in percentage of reactions present in tumor vs. normal tissues.

Additionally, tumors overexpress fatty-acid synthase (*FASN*) and undergo significant *de novo* fatty-acid synthesis [34]—*FASN* has been identified as a drug target in many tumors [35]. Fatty acid activation reactions are catalyzed by acyl-CoA synthetase (*ACS*), which acts downstream of FASN and converts long-chain fatty acids to acyl-CoA. Fatty acid activation is a critical step in several lipid metabolic pathways, including phospholipid and triacylglycerol biosynthesis. Some genes in this pathway (e.g., *ACSL4* and *ACSL5*) are overexpressed in certain types of cancer and inhibition of these genes induced apoptosis in cancer cells [36]. Notably, eicosanoid metabolism is the second most tumor-enriched pathway. Eicosanoids, which are biologically active lipids derived from arachdonic acid by cyclooxygenase, lipoxygenase, and P450 epoxygenase, have been implicated in inflammation and cancer [37]. Biologically active sphingolipids are involved in cancer pathogenesis-ceramide functions as a tumor-suppressor lipid, while sphingosine-1-phosphate functions as a tumor-promoting lipid [38]. This supports the identification of the sphingolipid pathway as enriched in tumor metabolic networks.

As a comparison, we also calculated the enrichment statistic for these brain and tumor enriched pathways using only expression data. Using the gene-reaction-pathway annotation from *Recon 1*, we calculated the average ubiquity score of metabolic genes in a pathway (i.e., how often the gene is expressed in tissue samples) and compared values for the above pathways in brain vs. non-brain and tumor vs. normal tissues. As shown in Additional file 3: Table S12, only 4 of the 10 brain-enriched pathways and 1 of the 9 tumor-enriched pathways identified by the model-based approach are also found by expression data alone, respectively. This shows the increased signal that can be extracted through the model-based approach.

**Figure 5:**
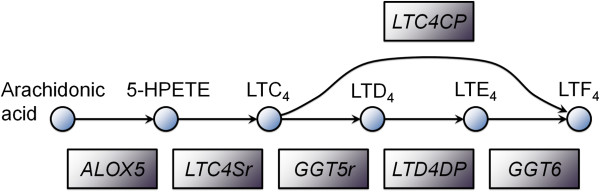
**The leukotriene synthesis pathway formed by the reactions occur significantly more often in 17 tumor tissues compared to corresponding normal tissues.** 6 reactions are shown; the other 7 reactions transport metabolites between cellular compartments.

We repeated the analysis to identify *individual reactions* that occur significantly more frequently in tumor tissue models than in normal tissues. Interestingly, the top most differentially included reactions (Table 5) together form part of the eicosanoid metabolism pathway, from arachidonic acid to leukotriene A4, C4, D4, E4 and F4 (Figure 5). The first two reactions are catalyzed by 5-lipoxygenase, which is induced by inflammatory stimuli and is often constitutively expressed in various cancers [37]. Furthermore, inhibition of 5-lipoxygenase has been shown to reduce cell proliferation and angiogenesis [39] and augment the antitumor activity of other drugs [40]. Leukotrienes have been implicated in various diseases such as asthma, cardiovascular diseases, and cancer [41]. For example, leukotriene C4 and D4 promote angiogenesis [42]; leukotriene D4 also promotes intestinal epithelial cell migration [43]. While involvement of genes and metabolites in the eicosanoid metabolic pathway has been reported in some cancers, our pathway and reaction level analysis revealed the importance of this pathway across a broad range of tumors arising from many different tissues.

**Table 5:** Top 13 reactions over-represented in tumor tissue models versus corresponding normal tissue models

**Reactions**^**a**^	**% tumor models**	**% normal models**	**Rank sum p-value**
ALOX5	72%	6%	8.6E-05
ALOX52	72%	6%	8.6E-05
EX_leuktrC4(e)	72%	6%	8.6E-05
GGT5r	72%	6%	8.6E-05
GGT6	72%	6%	8.6E-05
GLUtr	72%	6%	8.6E-05
GTHRDtr	72%	6%	8.6E-05
LEUKTRA4tr	72%	6%	8.6E-05
LEUKTRC4t	72%	6%	8.6E-05
LEUKTRD4tr	72%	6%	8.6E-05
LTC4CP	72%	6%	8.6E-05
LTC4Sr	72%	6%	8.6E-05
LTD4DP	72%	6%	8.6E-05

^a^These reactions form a pathway that catalyzes the production of leukotrienes from arachidonic acid.

#### Comparison of TSEM kidney model with the existing kidney metabolic model

As a demonstration of the utility of models in TSEM, we compared the TSEM kidney (*kidney*TSEM) metabolic model with the existing reduced kidney metabolic model (*kidney*Reduced) from Chang *et al.*[12]. While *kidney*TSEM is a genome-scale metabolic model with 1416 reactions, *kidney*Reduced aims to capture the core kidney metabolic phenotype and only includes 443 reactions. First, we compared the 578 and 34 gene-associated reactions unique to each model that also have protein staining evidence (Additional file 3: Table S13). 554 and 32 of gene-associated reactions unique to each model have non-negative protein staining, respectively. As expected, the *kidney*TSEM model includes many more enzymes that are expressed in the kidney.

Chang *et al.* compiled a list of 41 important renal metabolic functions, which consisted of the secretion and uptake of metabolites that regulate blood pressure. Following Chang *et al.*, we added exchange and demand reactions for the 41 metabolites, and maximized the uptake or secretion of each metabolite according to whether it is absorbed or secreted by the kidney.

*kidney*TSEM was able to achieve 35 of the 41 renal metabolic functions (Additional file 3: Table S14) while *kidney*Reduced—as expected—achieved all 41 renal metabolic functions that it was designed for. The six renal functions that *kidney*TSEM failed to achieve included secretion of prostaglandin I2, vitamin D, tryptamine, and absorption of acetate, oxalate, and L-carnosine, demonstrating a need for additional manual curation after the automated reconstruction.

Chang *et al.* also compiled a list of 20 gene deficiencies that are known to cause kidney disorders. 11 of the 20 genes are in *kidney*TSEM model, and all 11 gene deficiencies were predicted by *kidney*TESM to affect at least one of the 35 renal metabolic functions *kidney*TSEM can achieve under normal conditions (Additional file 3: Table S15). Thus, the recall rate was 55%, with a precision of 100%. Although not as accurate as *kidney*Reduced, *kidney*TSEM can simulate most renal metabolic functions and demonstrates good predictability of genetic perturbations. It is important to note that while *kidney*Reduced is based on a significant amount of metabolomics data, transcriptomic data, and most importantly, manual literature curation, the reconstruction of *kidney*TSEM is fully automated using transcriptomic data alone – an important point for rapid applicability to a broad range of contexts.

### Comparing mCADRE with the recently published INIT method

During the preparation of this manuscript, a new method (Integrative Network Inference for Tissues, INIT) capable of genome scale tissue-specific metabolic network reconstruction was published. This method was used to build genome-scale metabolic networks for 69 human cell types and 16 cancer types, collectively referred to as the Human Metabolic Atlas (HMA) [44]. The fundamental strategy of mCADRE and INIT is somewhat similar. Both mCADRE and INIT start from a generic human metabolic model, and use expression data to infer a tissue-specific sub-network. Both methods require that the model should be able to produce certain important metabolites. While mCADRE requires the model to produce universally important metabolites from simple precursors like glucose (which may overestimate the metabolic capabilities of human cells), INIT allows the model to uptake all metabolites to produce these key metabolites (which may underestimate the metabolic capabilities of human cells).

There are certain important differences between the two methods. INIT primarily uses the evidence from the Human Protein Atlas (HPA) as input, but can also incorporate gene expression and metabolomics data; algorithm parameters (e.g., weights assigned to different level of evidence) are also optimized for HPA data [44]. mCADRE can use any data that can quantitatively measure mRNA or protein abundance, but herein primarily uses gene expression microarray data. Although proteome data from HPA provide more direct evidence for the existence of corresponding metabolic reactions, current proteomic data is much less comprehensive than transcriptomic data from microarray or RNA-seq. To date, transcriptomic data from public repositories have explored a much broader range of human tissue/cell types and pathophysiological conditions: we were able to use mCADRE to build 126 human tissue-specific metabolic models based on transcriptomic data from a single microarray platform. Additionally, while mCADRE and other model building algorithms (MBA, GIMME, iMAT, etc.) are based on the steady state assumption of no net accumulation of metabolites, i.e., mass-balance, INIT allows for a small positive net accumulation of metabolites if a metabolite is present in a cell type according to metabolomics data.

We validated mCADRE by showing that the mCADRE-built liver model can simulate a wide range of liver metabolic functions (Table 2 and Additional file 3: Table S6-S8). An INIT-built liver model was shown to more accurately cover liver metabolic gene expression than the manually reconstructed HepatoNet1 [13]. However, it is unclear how this better coverage at the gene level will translate to model functionality: while HepatoNet1 was tested to be able to simulate a comprehensive set of 442 liver metabolic objectives, no such metabolic function simulation was reported for the INIT-built liver model.

We also compared the Tissue-Specific Encyclopedia of Metabolism (TSEM) built by mCADRE and the Human Metabolic Atlas built by INIT. Overall, TSEM included 126 models and HMA included 85 models. HMA included 16 cancer models while TSEM included 26 cancer models; 11 are shared. There are 100 and 69 normal tissue/cell types in TSEM and HMA respectively. Overall, the two collections shared 21 normal tissues (counting multiple cell types of the same tissue in HMA as one single tissue), with 30 unique to HMA and 79 to TSEM. One main difference is the coverage of tissues in the brain. While HMA included 8 models covering 3 brain structures (each brain structure has 2 or 3 cell-type specific models), TSEM included 30 models covering 30 distinct brain structures. Thus, the two collections of tissue-specific metabolic models are largely complimentary, with TSEM covering many more tissues.

## Conclusion

Large amounts of data have accumulated in public data repositories, characterizing the molecular phenotype of a variety of human tissue and cell types across a wide range of pathophysiological states [45]. However, the number of available tissue-specific metabolic models, which enable the systematic simulation of metabolic functions in normal and disease contexts, remains relatively small. To bridge this gap, we have developed a new automated method (metabolic Context specificity Assessed by Deterministic Reaction Evaluation, mCADRE) to efficiently build tissue-specific metabolic models in a high-throughput manner. From the comparison of brain and non-brain tissue models and the comparison of tumor and normal tissue models, it is clear that the pathway-level analysis is in agreement with literature. The corresponding models therefore enable further exploration of brain-specific metabolic functions and identification of drug targets that specifically kill tumor cells with minimal side effects on normal tissues. Combined with automated data acquisition and annotation tools [46,47], mCADRE has the potential to transform large repositories of gene expression data into repositories of functional tissue-specific metabolic models.

Importantly, metabolism is under extensive transcriptional regulation [48]. Mutations in transcription factors can cause various metabolic diseases [49], and many tumor suppressor genes and oncogenes are also transcriptional regulators of metabolism [50]. Combined with methods that automatically integrate transcriptional regulatory networks and metabolic networks [51], mCADRE may help to systematically identify the metabolic effects of transcription factors perturbations in various tissues. Ultimately, we hope to expand the TSEM to include both metabolic and corresponding transcriptional regulatory networks for many tissues and cell types. Additionally, metabolic interactions *between* different tissues and cell types play important roles in health and disease [52-55], and there have been pioneering studies that used integrated multi-cell type or multi-tissue type models to such interactions [18,19]. The large collection of tissue and cell type specific models in the TSEM may facilitate the integrated modeling of metabolic interactions such as those between adipocytes and macrophages, different brain tissues, and between tumor and stromal microenvironment.

## Methods

The majority of the automated reconstruction pipeline in mCADRE, including the MAS5 detection call, is implemented in Matlab, and the pipeline produces genome-scale draft metabolic models from raw expression intensity files. To validate this pipeline, we built a liver model with MAS5 as the binarization method and compared it to a liver model constructed with MBA. Aside from the hepatic functional testing of liver models, all steps described below were subsequently applied to generate context-specific models for 126 different human tissues. Note that the Gene Expression Barcode project already used the barcode method to produce binarized transcriptomic data for the 126 tissues, so MAS5 was not used in this case. As the barcode binarization tends to be more stringent in calling a gene expressed than MAS5 [20], the resulting models may also be smaller than when MAS5 is used.

### Gene expression data processing

This new method uses gene expression microarray data as input evidence to prune a generic model (e.g., *Human Recon 1*) to a context-specific subset; the SBML file for *Recon 1* was obtained from the BiGG database [56] and converted into COBRA Toolbox [57] model structure for subsequent analysis. To construct a context-specific metabolic model for the liver, we acquired raw gene expression profiles from 23 liver tissue samples from [58-61] and GSE7307 (no citation available). All of these studies were identified and annotated by the Gene Expression Barcode Project [20]. To approximate the presence or absence of the enzyme and transporter-encoding gene in a particular profile, we used the Affymetrix MAS5 detection call to binarize raw microarray data[62]: present calls are treated as 1, while marginal and absent calls are treated as 0. Other binarization methods, such as the gene expression barcode [20], can also be used. The final binarized expression data for all genes *g*∈*G* in samples *n*∈*N* for a selected context or phenotype is represented as the expression matrix *X*^|*G*|×|*N*|^, where *X*_*g*,*n*_ = {0,1} represents the presence of gene *g* in sample *n*; for our liver training data, |*G*| = 20,283. There are 54,613 probe sets on the Affymetrix U133Plus2 platform (excluding quality control probes). Only probes that can uniquely map to a single gene are retained; these probes map to 20,283 unique genes. When multiple probes map to the same gene, the maximum expression value is used.

### Assigning evidence scores to reactions

For each reaction *r*∈*R* in the generic model, we assign evidence scores (***E***(*r*)) to deterministically evaluate which reactions to keep or remove when pruning to get a context-specific network. We first calculate the *expression-based evidence****E***_***x***_(*r*) for all reactions to provide an overall ranking and to divide reactions into core and non-core sets. Next, the network topology of the generic model is used to calculate the *connectivity-based evidence****E***_***c***_(*r*) for each non-core reaction; this provides a second level of evidence when determining the order of reactions to remove during pruning. Finally, if expression- and connectivity-based evidence is insufficient to determine the rank of a reaction, *evidence based on confidence level* in the generic model ***E***_*l*_(*r*) is considered.

#### Expression-based evidence

After binarizing the input data, we first quantify how often a gene is expressed across samples of the same context; this is the ubiquity score *U*(*g*) for each gene *g*:

$$U\left(g\right)=\left(1/|N|\right){\sum_{n}}_{\in N}{X_{g}}_{,n}.$$

This score ranges from 0 (not expressed in any context samples) to 1 (ubiquitously expressed in context samples). According to gene-reaction rules, ubiquity scores for metabolic genes are mapped to corresponding reactions. That is, the expression-based evidence ***E***_***x***_(*r*) for reaction *r* is a function of how often its associated genes *g*_*r*_∈*G*_*r*_ are expressed in the selected context, as measured by the ubiquity score:

$$E_{x}\left(r\right)=f\left(U\left(g_{r}\right)\right),g_{r}\in G_{r}.$$

The relationship between the ubiquity scores of *G*_*r*_ and ***E***_***x***_(*r*), denoted by *f*, is a composite of the Boolean gene-reaction rules defined in the generic model: AND is replaced with MIN, while OR is replaced with MAX, following ref [17] (Figure 1A). By definition, the expression-based evidence ***E***_***x***_(*r*) also ranges from 0 to 1, indicating how likely the reaction is to be present in the selected context. The high-confidence core set of reactions is then defined as those with ***E***_***x***_(*r*) > 0.9 when building *liver*CADRE with MAS5 call binarization, and ***E***_***x***_(*r*) > 0.5 when building the 126 tissue models with the Barcode binarization. Higher cutoff is used for MAS5 call binarized data, as it is less stringent than Barcode in calling a gene expressed. Reactions with ***E***_***x***_(*r*) = 0 are defined as the negative reaction set: these reactions have strong evidence of not being active in the tissue context.

#### Connectivity-based evidence

For non-core reactions, we use network topology to define a secondary metric called *connectivity-based evidence****E***_***c***_(*r*). This score is particularly designed to rank non-gene-associated reactions, which account for 40% of all reactions in *Recon 1*, and by definition, will not be in the core because they are not associated with expression data. The connectivity-based evidence for each non-core reaction accounts for both the expression-based evidence and connectedness of all adjacent reactions (core or non-core). Using the stoichiometric relationships defined in the *S* matrix, we can describe whether any two reactions in the generic model are connected (i.e., share at least one metabolite) with the binary *adjacency* matrix *A*^|*R*|×|*R*|^. Specifically, *A*_*i*,*j*_ = {0,1}, where 1 indicates reaction *i* is connected to reaction *j*.

We consider the outgoing *influence I*(*r*) of each reaction as its normalized connectedness to all adjacent reactions. That is, for each reaction *r*,

$$I\left(r\right)=1/{\sum_{j}}_{\in R/r}\left({A_{r}}_{,j}\right).$$

In this way, *r* exhibits influence on all other reactions *j*∈*R*/*r* that is inversely proportional to the number of reactions to which it is connected. Furthermore, we measure the *weighted influence WI*(*r*) = ***E***_***x***_(*r*)×*I*(*r*) such that *r* will exhibit stronger influence on connected reactions if it was found to have strong expression-based evidence; reactions with ***E***_***x***_(*r*) = 0 thus have no weighted influence on adjacent reactions.

Finally, we define connectivity-based evidence ***E***_***c***_(*r*) as the net incoming weighted influence to reaction *r* from all other reactions *j*∈*R*/*r*:

$$E_{c}\left(r\right)={\sum_{j}}_{\in R/r}\left(\mathrm{WI}\left(j\right)|{A_{r}}_{,j}=1\right).$$

If a non-core reaction *r*_*j*_ is connected to a highly expressed reaction *r*_*i*_ that has few other connections, this provides strong support for its inclusion in the context-specific model. Conversely, if a core reaction *r*_*i*_ is connected to many other reactions, then it is less clear whether any particular connected non-core reaction *r*_*j*_ is the one that functions in a pathway with *r*_*i*_ in the pruned network; as such, the resulting connectivity-based evidence for *r*_*j*_ will be lower.

#### Confidence level-based evidence

Confidence scores indicate the level of biological evidence associated with each reaction, as determined during manual curation of the generic metabolic model—in this case, *Human Recon 1*. The confidence level evidence ***E***_*l*_(*r*) for a reaction ranges from 1 (*in silico* modeling evidence only) to 3 (experimental biochemical or genetic evidence); midlevel scores (2) indicate some physiological evidence, or experimental support from a related organism, and a score of 0 indicates that the reaction was not evaluated. Importantly, these confidence scores represent evidence for the generic model, not for the specific context, and thus are considered as a tertiary measure of evidence for non-core reactions.

### Pruning the generic model

After defining the high-confidence core and ranking all non-core reactions, our algorithm attempts to sequentially remove each non-core reaction, starting from those ranked at the bottom (lowest evidence). The selected reaction will be removed only if (i) the core set of reaction remains consistent; and (ii) removal does not prevent model from producing any key metabolites. Reactions in high-confidence core set can only be removed when (i) reactions in the negative reaction set (reactions with ***E***_***x***_(*r*) =0) are needed to enable flux through the high confidence core reactions; (ii) by removing the high confidence core reactions, more non-core reactions (including those in the negative reaction set) will be removed. Consistency of the core reaction set is confirmed by calculating the maximum and minimum flux for each reaction, and ensuring that at least one is non-zero. As the naïve implementation of flux variability analysis (FVA) is extremely slow, we adapted the *checkModelConsistency* module described by Jerby *et al.* in [14] for optimal performance in Matlab—in particular, we included the option to use the efficient fastFVA algorithm [27].

The list of key metabolites that must be produced from glucose is compiled based on the universal metabolic model validation test in [18]. This includes metabolites in glycolysis, TCA cycle, pentose phosphate pathway, as well as non-essential amino acids, nucleotides, palmital-CoA, cholesterol, and several membrane lipids. A full list of these key metabolites is in Additional file 3: Table S1. Instead of testing the production of all non-essential fatty acids, as in [18], we only tested the production of palmital-CoA, which is derived from palmitate, the first fatty acid produced in fatty acid synthesis, and the precursor of longer chain fatty acids. Similarly, we only tested those membrane lipids that can be derived from glucose and non-essential amino acids. With the addition of essential nutrients like choline, these membrane lipids can be transformed to other membrane lipids such as phosphatidylcholine and sphingomyelin that cannot be directly synthesized from glucose. We only check the production of pyrimidine nucleotides from glucose, as *de novo* pyrimidine synthesis can occur in a variety of tissues [22]. As *de novo* purine synthesis occurs primarily in the liver and other tissues use the salvage pathway [22], we test the ability of all tissues to synthesize purine nucleotides from purines bases and 5-phosphoribosyl 1-pyrophophate (PRPP).

### Functional test of liver models

In the amino acid degradation test, the model is only allowed to uptake glucose and the amino acid being tested; all other organic metabolites are constrained to be efflux only. Transport of inorganic compounds (oxygen, carbon dioxide, water, etc.) is unconstrained, except ammonia: as ammonia detoxification is an important hepatic function, only ammonia influx is allowed. The simulation objective function is to maximize the uptake of the amino acid being tested. Using FVA, if the model can allow for finite urea efflux *and* amino acid influx, the amino acid degradation test is declared as passed.

Similarly, in the ammonia detoxification test, only glucose uptake is allowed, and the objective is to maximize ammonia uptake. This test is passed if the model can allow for finite ammonia influx *and* urea efflux. The ethanol detoxification test is the same as ammonia detoxification test, except that no urea efflux is required and ethanol is constrained to be influx.

In the glucogenic test, the model is only allowed to uptake the glucogenic substrate being tested while all other organic compounds, including glucose, are constrained to be efflux only. Ammonia is only allowed to be influx, and urea is only allowed to be efflux. The simulation objective is to maximize glucose secretion. This test is passed if the model can allow for finite glucose efflux. Glucogenic substrates tested are the 18 glucogenic amino acids (all 20 amino acid except leucine and lysine, which are exclusively ketogenic), lactate, pyruvate, and glycerol.

In growth simulation, the widely-used RPMI-1640 tissue culture medium was used. Detail of medium composition is in Additional file 3: Table S11. The biomass equation was adopted from [14]. The full list of biomass components is in Additional file 3: Table S10. It consists of amino acids, nucleotides, deoxynucleotides, lipids etc. *Recon 1* lacks a reaction accounting for the formation of glycogenin, the primer for glycogen synthesis, so a sink reaction for glycogenin is added to all the liver models to allow for glycogen synthesis.

## Competing interest

The authors declare that they have no competing interests.

## Authors’ contributions

YW and NDP designed research; YW and JAE performed research; YW, JAE, and NDP analyzed data; and YW, JAE, and NDP wrote the paper. All authors read and approved the final manuscript.

## Supplementary Material

Additional file 1 Codes and input data to run mCADRE in a zipped file. Click here for file

Additional file 2 All 126 TSEM metabolic model files in a zipped file. Click here for file

Additional file 3: Table S1 Includes a list of universally important metabolites that must be produced from glucose. **Table S2.** Includes a sensitivity analysis of the inactivated core to non-core ratio. **Table S3.** Includes GEO accession numbers for microarray samples used to build *liver*CADRE. **Table S4.** Includes the coverage and functionality comparison of *liver*CADRE, the original liver, MBA model, and the new liver MBA model (based on the same training data as *liver*CADRE). **Table S5.** Includes the GEO accession numbers for microarray samples used in independent comparison of *liver*CADRE and *liver*MBA. **Table S6.** Includes the Human Protein Atlas staining data for 560 metabolic genes. **Tables S7-S9.** Include the detail results of liver metabolic function tests. **Table S10.** Includes the biomass composition. **Table S11.** Includes the RPMI-1640 medium. **Table S12.** Includes the enrichment of pathways listed in Table 3 and Table 4 in brain and tumor tissues based on gene expression data alone. **Table S13.** Includes comparison of protein staining evidence for unique gene associated reactions in *kidney*Reduced and *kidney*TSEM. **Table S14.** Includes *kidney*TSEM simulation results of 41 renal metabolic functions. **Table S15.** Includes *kidney*TSEM simulation of effects of genetic perturbations on renal metabolic functions. Click here for file

Additional file 4 Inconsistent reactions in each of the 126 TSEM models. Click here for file

Additional file 5 liverCADRE in SBML format. Click here for file
